# Effects of Education, Nutrition, and Psychology on Preventing the Female Athlete Triad

**DOI:** 10.7759/cureus.55380

**Published:** 2024-03-02

**Authors:** Bijal Patel, Nicole Schneider, Pradeep Vanguri, Lailah Issac

**Affiliations:** 1 Osteopathic Medicine, Nova Southeastern University Dr. Kiran C. Patel College of Osteopathic Medicine, Davie, USA; 2 Health and Human Performance, Nova Southeastern University Dr. Kiran C. Patel College of Osteopathic Medicine, Davie, USA; 3 Sports Medicine, Nova Southeastern University Dr. Kiran C. Patel College of Osteopathic Medicine, Davie, USA

**Keywords:** menstrual dysfunction, psychology, nutrition, education, female athlete triad

## Abstract

The female athlete triad is a syndrome occurring in young female athletes defined by menstrual dysfunction, decreased energy availability (EA), and low bone mineral density (BMD). Although the triad includes these three conditions, not all three need to occur simultaneously for the diagnosis to be made. The goal of this review is to analyze published research on the female athlete triad and determine prevention methods in athletics. A review of 23 published sources using the PubMed database identified key recommendations, including education resources, psychological factors, and nutrition. It is recommended that athletes, parents, coaches, and healthcare professionals should learn about the risk factors, warning signs, and diagnosis for better prevention. Research revealed that eating disorders, self-esteem issues, and coach-athlete relationships should be evaluated and potentially managed with counseling. Finally, nutritional recommendations included maintaining EA, providing nutritional counseling, and proper nutritional education. Early intervention with proper education, psychological support, and nutritional management are vital to preventing the onset of the female athlete triad.

## Introduction and background

The female athlete triad includes menstrual dysregulation, low energy availability (EA), and a decrease in bone mineral density (BMD) [1]. In 1992, the female athlete triad included disordered eating, amenorrhea, and osteoporosis; however, in 2007, the definition was changed to EA, menstrual function, and BMD [2]. This syndrome is most common in active young women who participate in sports, most commonly endurance sports [2]. Energy availability is defined as the amount of caloric energy available from nutrition after taking account of the amount of energy spent from exercise [1]. Low EA could be due to an eating disorder, but it is not required for the diagnosis of the triad [1]. BMD is the measurement of the amount of minerals, such as calcium and phosphorus within the bone, and it is used as a tool to measure bone strength [1].

Eating disorders and low BMD have become a leading cause of the female athlete triad [2]. Most athletes will experience the triad at various intervals or be affected by one or two aspects of the triad [2]. Menstrual dysregulation includes amenorrhea, oligomenorrhea, or other menstrual irregularities. Amenorrhea is defined as the lack of menses, which can be primary or secondary, either lacking menstruation by 15 years or ceasing of menstrual cycles after previously menstruating [2].

Due to the complexity of the triad, diagnosis and treatment are challenging and require an interdisciplinary team to help prevent serious complications from occurring in athletes. When it comes to the diagnostic tests for the triad, they include specific tests for each component: low EA, menstrual dysfunction, and low BMD. For example, a low EA would involve a complete blood count, metabolic panel, and phosphorus and magnesium testing [1]. Menstrual dysfunction diagnostic testing would involve urine human chorionic gonadotropin, follicle-stimulating hormone, thyroid-stimulating hormone, free thyroxine, and prolactin, to name a few [1]. A dual-energy X-ray absorptiometry would be utilized when diagnosing low BMD [1]. This team must have a level of understanding of the disorder from the education, nutrition, and psychological aspects. In addition to healthcare professionals, athletes, coaches, and family members should partake in understanding the disorder in order to prevent it from occurring.

This research is a literature review that investigates previous studies about the triad discovered in various female athletic sports. We explore how to improve the diagnosis and treatment of the triad in female athletes through an emphasis on education, psychology, and nutrition.

## Review

Methods

**Figure 1:**
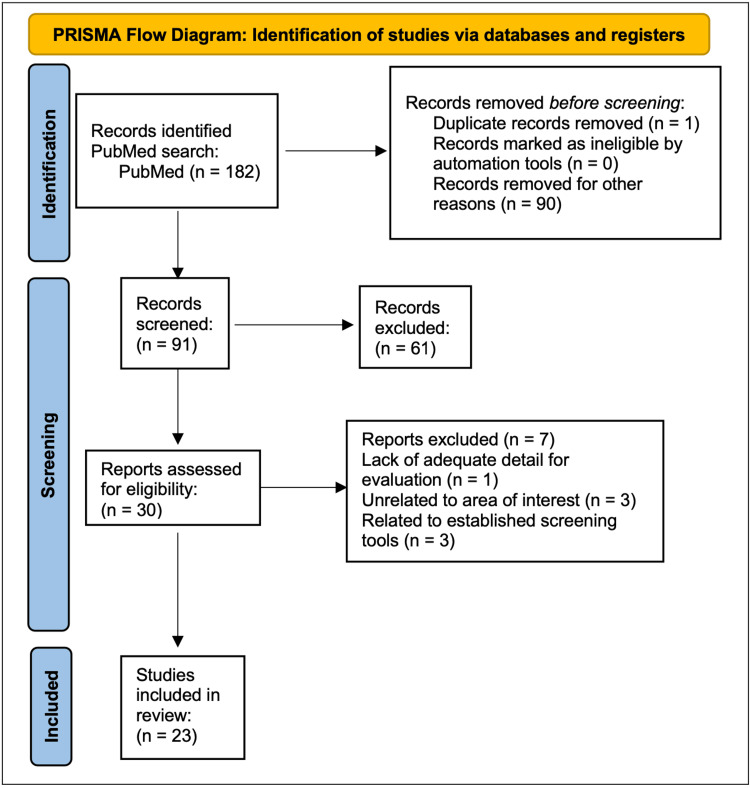
PRISMA flow diagram: identification of studies via databases and registers PRISMA: Preferred Reporting Items for Systematic Reviews and Meta-Analyses

This literature review analyzed current studies to determine the best prevention strategies through education, psychology, and nutrition. A comprehensive search on PubMed was performed with the search terms “female athlete triad” AND “training,” where 182 articles were discovered. Then, the articles were filtered to only include from years 2014 to 2022, limiting the articles to 91. Out of the 91 articles, an exclusion of secondary articles, male athletes, and incorrect studies resulted in 30 articles and included primary articles, articles in the English language, and articles regarding the female athlete triad. Of those 30 articles, seven were removed for lacking adequate results for evaluation, unrelated to the area of interest, and lacking established screening tools. Twenty-three articles were then analyzed for methods to prevent the triad, which resulted in the themes of utilizing education, psychology, and nutrition. Identification, screening, and eligibility of articles were conducted following Preferred Reporting Items for Systematic Reviews and Meta-Analyses (PRISMA) guidelines with the PRISMA flowchart included in Figure 1 [3]. Therefore, the three aspects were further examined to suggest methods of in-depth prevention strategies in young female athletes. 

Results

Education Epidemiology

A crucial aspect of diagnosing the triad early on includes educating the athletes, coaches, parents, and healthcare professionals. Education about aspects of the triad including classifications, risk factors, warning signs, and approach to diagnosis is vital to prevention efforts. While athlete awareness is the primary goal of preventing the triad, support staff awareness is also necessary to better assist the athletes in a healthier lifestyle.

Three aspects that make up the triad are menstrual dysregulation; low EA, which may or may not be due to an eating disorder; and low BMD [1]. There are many risk factors that healthcare professionals, coaches, parents, and athletes should monitor. Lean sports are ones that prioritize weight or “thinness,” which are some of the highest-risk sports that could lead to the triad [2]. Lean sports can be broken down into categories: aesthetic and endurance sports. Aesthetic sports emphasize body image, such as diving, ballet, cheerleading, and gymnastics [2]. Endurance sports utilize repetitive isotonic movements of large muscle groups; examples include distance running, swimming, and cycling [2]. Some risk factors that are crucial to recognize and address are eating disorders; age with the understanding that this triad can also occur in women greater than 35 years of age with a history of eating disorders, which may be an exception; menstrual dysfunction due to low body mass index (BMI); lean or aesthetic sports; stress fractures due to low BMD; and menstrual irregularities [2,4]. Warning signs are also important to consider, including appearance dissatisfaction, fracture history, mood changes, weight loss, decline in performance, and frequency of illnesses [2]. Therefore, athletes should seek regular checkups with primary care physicians, who can screen for these risk factors that could lead to a decline in performance, mood changes, dramatic weight loss, amenorrhea, and recurrent injuries, such as multiple stress fractures [1].

Table 1 addresses the key points in the literature related to the education of the female athlete triad.

**Table 1:** Summary points of education-related articles RED-S: Relative energy deficiency in sport; EA: energy availability

Author(s), year	Title	Summary Points
Rauh et al., 2014 [5]	Associations Between the Female Athlete Triad and Injury Among High School Runners	Educating high school runners, their coaches, parents, and healthcare professionals could allow for a reduction in injuries by encouraging healthier diets and training.
Brown et al., 2020 [6]	Increased Female Athlete Triad Knowledge Among Collegiate Dancers Following a Brief Educational Video Intervention	There is a lack of knowledge on the triad in dancers; therefore, educational videos could have an impact on the understanding of the triad.
Gram et al., 2021 [7]	Injuries and Illnesses Among Competitive Norwegian Rhythmic Gymnasts During Preseason: A Prospective Cohort Study of Prevalence, Incidence and Risk Factors	Early prevention programs can reduce the risk of overuse injuries in non-menstruating female Norwegian rhythmic gymnasts.
Lodge et al., 2022 [8]	Knowledge of Triad and RED-S in Female Cross-Country Athletes and Support Staff	The female cross-country athletes had the lowest knowledge about the triad and RED-S; therefore, collegiate institutions should implement policies about the risks of the triad and RED-S.
Cheng et al., 2021 [9]	Menstrual Irregularity, Hormonal Contraceptive Use, and Bone Stress Injuries in Collegiate Female Athletes in the United States	Hormonal contraceptive use can mask other causes of menstrual irregularities in athletes perpetuating misdiagnosis and stress fractures; therefore, education on side effects is necessary.
Dimitriou et al., 2014 [10]	Bone Mineral Density, Rib Pain and Other Features of the Female Athlete Triad in Elite Lightweight Rowers	Lightweight rowers who restrict their diet for intentional weight loss leading to energy deficiencies and lower bone mineral densities would benefit from education.
Barrack et al., 2021 [11]	Disordered Eating, Development of Menstrual Irregularity, and Reduced Bone Mass Change After a 3 Year Follow-Up in Female Adolescent Endurance Runners	Education may decrease the risk of the triad and physiological/psychological disorders in athletes with misconception of body weight.
Torres-McGehee et al., 2021 [12]	Energy Availability with or without Eating Disorder Risk in Collegiate Female Athletes and Performing Artists	Sports with a higher focus on food restriction would benefit from counseling and education.
Smith et al., 2022 [13]	Examination of the Prevalence of Female Athlete Triad Components Among Competitive Cheerleaders	Competitive cheerleaders experience low EA; therefore, nutritional education and knowledgeable coaches can help prevent the triad.
Nguyen et al., 2014 [14]	Osteoporosis Health Beliefs of Women With Increased Risk of the Female Athlete Triad	Implementation of education about osteoporosis and intense training could prevent injuries.
Folscher et al., 2015 [15]	Ultra-Marathon Athletes at Risk for the Female Athlete Triad	Education is necessary for endurance athletes due to lack of knowledge to inform them of the risks and ways to prevent the triad.

Psychiatric Epidemiology 

It is important to discuss the psychological impact of the triad. Most young women are susceptible to the development of eating disorders, depression, and self-esteem issues, especially during adolescence [11]. However, athletes who are under much more scrutiny and pressure seem to be at increased risk. Athletes who partake in sports that prioritize weight and thinness have greater incidences of disordered eating [11]. The potential outcomes of those unhealthy behaviors result in physiological derangements, such as oligomenorrhea, lower BMD, and bone stress injuries, such as stress fractures [10]. These findings are concerning and worrisome, more specifically for those who continue sports participation without psychological support.

Walsh et al. determined that active lightweight rowers were more likely at risk for pathologic behaviors, such as food restriction, binge eating episodes, vomiting, and laxative abuse [16]. The athletes reported a significant amount of worry and stress regarding the amount of food and calories consumed [16]. This article also emphasizes the importance of stress and injuries on an athlete. Athletes who have a much more dedicated and ingrained perception of their athletic role in their identity result in a much more detrimental response to their psyche when it comes to injury [16]. The implications include loss of confidence and fear of losing the role as an athlete or a vital team member and their self-esteem [16]. The subsequent psychological response can be more injurious to the athlete than the injury itself.

Carson et al. evaluated athlete-coach relationships to investigate any malignant behaviors and their relation to any psychological issues [17]. Female National Collegiate Athletic Association Division 1 (NCAA D1) distance runners were interviewed, and their responses revealed the complicated relationships with their coaches and the stigma of body image issues that comes with being a distance runner [17]. Table 2 provides a summary of the key points of the research related to psychology.

**Table 2:** Summary points of psychology-related articles NCAA D1: National Collegiate Athletic Association Division 1

Author(s), Year	Title	Summary Points
Walsh et al., 2020 [16]	Exploring Health Demographics of Female Collegiate Rowers	Collegiate lightweight rowers having weight requirements to meet in order to compete increases the risk of developing disordered eating.
Carson et al., 2021 [17]	Culture and Environmental Associations With Body Image, Diet and Well-Being in NCAA D1 Female Distance Runners: A Qualitative Analysis	Coach-athlete relationships play a pivotal role in weight, psychological and athlete dynamics in female D1 athletes.

Nutrition

The most important factor in preventing the triad is to understand EA and the energy requirements of the athlete to avoid the consequences of unmet energy needs. EA in female athletes is calculated using female energy intake (caloric intake), energy expenditure, and the amount of energy available during training [18]. Caloric intake of less than 30 Kcal/kg x free fat mass/day led to derangements in the female menstrual cycle [19]. Consistently eating less than 30 Kcal/kg x free fat mass/day while training with high intensity leads to significant multisystem changes in metabolism. Athletes who are chronically deficient have led to physiological changes related to the thyroid, leptin levels, and female reproductive system [18]. Intense training stimulates a sympathetic response that is anorexigenic. Long-term training blunts the body’s normal response that is sensitized to detecting energy needs and when the body should be fed [18]. After a time, lower EA results in a lower metabolic resting rate and a higher risk of developing the female athlete triad [20]. Energy restriction suppresses the hypothalamic-gonadotropic axis and ovarian suppression presents as amenorrhea or oligomenorrhea. Furthermore, energy deficiencies affect leptin levels that can perpetuate female sex hormone suppression and menstrual dysfunctions [16].

Most athletes are unaware of the long-term complications of these dysfunctional behaviors and often lack nutritional access or knowledge to properly feed themselves to prevent energy deficiency [16]. Nutritional support is often not integrated into collegiate athlete training. Competitive collegiate athletes were analyzed for components of the triad. These athletes were found to be eating less than 30 Kcal/kg x free fat mass/day and practicing intentionally or unintentionally undereating and pathologic eating behaviors [12,13]. Female cheerleaders were surveyed, and most did not have access to proper nutritional education or coaches who were not equipped with the knowledge of the nutrient demand of the cheerleading sport [13]. Dimitriou et al. discovered not many active athletes were given proper nutritional education when compared to retired athletes, and it is unknown as to why this occurs [10].

Nutritional coaching and its efficacy were reviewed by several studies in this review. Syed et al. conducted a case report to determine the efficacy of the nutritional program on an athlete. The results showed the program was therapeutic in resolving the female athlete triad [21]. The program was able to help maintain BMD and body fat percentage long-term in athletes experiencing significant weight loss [21]. A female triathlete who was observed for her inter-day and intra-day caloric intake was unable to consume enough calories to balance the amount that was spent during training [22]. Most of the training sessions were done in a caloric deficit due to factors, such as fullness while training [22]. The athlete would eventually eat almost 46% of her calories after her training sessions. It was reasoned that the athlete’s high-protein diet resulted in earlier satiety and decreased food intake [22]. Table 3 identifies areas of the literature specific to nutrition and the female athlete triad. 

**Table 3:** Summary points of nutrition-related articles EA: energy availability; BMD: bone mineral density; LEAF: low energy availability in females

Author(s), year	Title	Summary points
Schaal et al., 2021 [18]	Decreased Energy Availability During Training Overload Is Associated With Non-functional Overreaching and Suppressed Ovarian Function in Female Runners	Consistent low caloric intake and available energy can result in endocrine abnormalities affecting leptin levels, thyroid hormone levels, and core body temperature.
Areta, 2020 [19]	Case Study: Resumption of Eumenorrhea in Parallel With High Training Load After 4 Years of Menstrual Dysfunction: A 5-Year Follow-Up of an Elite Female Cyclist	Consistently low EA leads to menstrual derangements resulting in restoration of menses by increasing caloric intake taking up to six months.
Hooper et al., 2021 [20]	Performance and Health Decrements Associated With Relative Energy Deficiency in Sport for Division I Women Athletes During a Collegiate Cross-Country Season: A Case Series	Nutritional coaching by a registered team dietician that encouraged increased EA increasing resting metabolic rate (RMR) overall and better performance.
Syed-abdul et al., 2018 [21]	Impact of a Professional Nutrition Program on a Female Cross Country Collegiate Athlete: A Case Report	Sports emphasizing appearance and intense training schedules show lower BMD and menstrual cycle problems.
Vescovi et al., 2016 [22]	Case Study: Impact of Inter and Intraday Energy Parameters on Bone Health, Menstrual Function, and Hormones in an Elite Junior Female Athlete	Menstrual dysfunction can result due to inconsistent EA and underconsumption of calories to compensate for energy expenditure.
Amorim et al., 2021 [23]	Associations between nutrition, energy expenditure and energy availability with bone mass acquisitions in dance students: A 3-year longitudinal study	Increasing EA can reduce injuries in female dancers due to increasing their bone mass density.
Ackerman et al., 2018 [24]	Low energy availability surrogates correlate with health and performance consequences of Relative Energy Deficiency in Sport	LEAF questionnaire was used to determine the relationship between decreased EA and BMI. The athletes with a higher BMI were found to have low EA.
Kamemoto et al., 2021 [25]	Relationship between weight management and menstrual status in female athletes: a cross-sectional survey	Athletes with weight loss had higher rates of menstrual dysfunctions and physical effects than those without weight loss.
Tenforde et al., 2018 [26]	Sport and Triad Risk Factors Influence Bone Mineral Density in Collegiate Athletes	BMD in non-endurance athletes was higher compared to those in endurance sports; Lower BMI correlated to lower BMD and menstrual changes.
Ikegami et al., 2022 [27]	The Influence of Low Energy Availability on Bone Mineral Density and Trabecular Bone Microarchitecture of Pubescent Female Athletes: A Preliminary Study	Low EA and low percent ideal body weight is related to decreased BMD in lumbar bone structure in growing teens.

Discussion

Education

Educating athletes and support staff on the risk factors and warning signs can help prevent the female athlete triad; however, proper diagnosis is key. Performing an evaluation that includes a thorough medical and menstrual history, skeletal health, family and psychosocial history, physical exam, and laboratory exam creates adequate background information necessary for diagnosis. During the physical exam, dry skin, lanugo, scars on knuckles, and enlargement of parotid glands should also be noted as these may potentially suggest an eating disorder, a key risk factor for the triad [2]. Hypoestrogenism may be seen during a pelvic exam showing vaginal atrophy or by performing laboratory tests [1]. Lab work and imaging studies can assist with eliminating other differential diagnoses prior to determining a female athlete triad diagnosis.

Overall, being aware of unique training, nutrition characteristics, and rapid weight loss can be crucial for the support staff of the athlete and help with early intervention [4]. For the athletes to feel secure, a positive relationship and skillful interviewing techniques are needed between a healthcare professional and an athlete [2]. Education about the triad is identified as crucial to preventing this disorder from occurring in young female athletes. Educating not only the athlete but the coaches, parents, and healthcare team can prevent serious injuries that can occur with this disorder.

*Psychology* 

The culture of sports and competitions is to succeed despite the impact that it can have on the individual athlete or the team dynamic [17]. The body image of runners emphasizes thinness and may affect the speed and performance of the runner [17]. Coaches are crucial to enforcing positive body image ideals upon the athletes, but some coaches may prioritize team performance over individual body image issues fostering a negative environment for disordered eating to occur [17]. Research revealed tactics, such as direct pressure, shame, and control of food and weight, to create an unhealthy and toxic environment [17]. These relationships may cause long-term outcomes on the athlete’s psyche, years after their career ends.

Understanding and recognizing psychological factors can help with preventing eating disorders and pathologic behaviors. This requires awareness and action by healthcare professionals, parents, cultures, and the athletes themselves. Athletes should be provided psychological support to address self-esteem, stressors, depression, and suicidal thoughts. Not only can the lack of caloric intake contribute to these psychological issues, but caloric intake can also be a risk factor for disordered eating. Furthermore, addressing athlete-coach relationships can also help with body image and well-being. Although not all athlete-coach relationships are detrimental, it is important for coaches to understand the long-term implications of their negative coaching strategies.

Nutrition

With the lack of proper support, there are more incidences and a higher likelihood of pathologic eating patterns, which include milder habits, such as dietary restrictions, undereating, insufficient protein intake, binging, and purging (laxative misuse and vomiting) [12]. To prevent pathologic eating patterns and subsequent complications, such as decreased BMD and rib stress fractures, professional nutritional support should be integrated into the training of the sport [10]. By increasing nutritional support and knowledge, female athletes are better prepared and able to facilitate their own nutritional needs. It is important to recognize that the female athlete triad is a result of chronic energy deficiency and that long-term injuries can be prevented by early diagnosis and management. Torres-McGehee et al. suggested athletes partaking in pathologic eating behaviors and with decreased energy intake would benefit from nutrition counseling and education with a dietician [12]. Interventions to restore menses mostly include increased EA and eating with a caloric surplus [19]. Just as the presentation of the triad is delayed, the resumption of menses is only possible over time, taking up to a day to over a year [19]. Restoration is only possible by consistently eating in a surplus and maintaining increased bone mass for several months [19].

Professional nutrition programs are helpful in assessing caloric demand with respect to their training. Having nutritional coaching and a registered team dietician who manages energy intake and EA has resulted in increased resting metabolic rate, better performance, and decreased injuries [20]. Nutritional coaching can help guide athletes to balance out the macronutrients in a manner that can properly fuel the athlete throughout the day and prevent issues with EA.

Certain nutrients are essential for maintaining proper bone and physical energy. Low ferritin levels, with ferritin being the stored form of iron, also have been implicated in affecting performance by causing extreme fatigue [20]. Maintaining ferritin levels about the threshold of 20 ng x mL^-1^ had statistically improved performances [20]. Reduction of vitamin D concentration has been a concern for athletes since it is suspected to be associated with lower BMD [20]. Therefore, by increasing calcium and vitamin D, athletes can maintain healthy bones, which in turn prevents osteoporosis [26]. A diet rich in fruits, vegetables, and high-energy carbohydrates is important for athletes [13]. Nutrition alongside weight gain over time is the best for addressing low BMI, nutritional deficiencies, bone health, and menstrual dysfunctions.

Strengths, Limitations, and Implications

This literature review analyzed articles regarding the female athlete triad and ways to prevent the triad from occurring. A strength of this article was the focus on three specific methods that could help with identifying the triad at an early stage through education, psychology, and nutrition. These three areas involve an approach to allow for a better understanding of the triad and the risks. Limitations to this study were the primary area of focus being on young female athletes rather than all athletes including males and females and all ages. The implication of this study is to stress the importance of knowledge about the female athlete triad in order to prevent female athletes from acquiring this syndrome.

## Conclusions

Young female athletes are susceptible to the triad for a multitude of factors. Female athletes undergoing high-intensity training in sports that emphasize the importance of thinness and weight are more likely at risk. Most athletes unfortunately are not educated in the long-term complications of the female athlete triad. The pressure and stress to fit the ideal body image for their sport encourage athletes to partake in pathologic eating behaviors. Lack of proper nutrition counseling and energy intake can lead to physiological derangements that result in menstrual dysfunction, osteoporosis, and low EA. For the early diagnosis and prevention of complications, female athletes and their coaches should receive proper education, appropriate nutrition, and psychological support.
